# Effect of morning versus night-time administration of proton pump inhibitor (pantoprazole) on thyroid function test in levothyroxine-treated primary hypothyroidism: a prospective cross-over study

**DOI:** 10.1186/s13044-023-00156-6

**Published:** 2023-06-01

**Authors:** Avivar Awasthi, Partha Pratim Chakraborty, Neeti Agrawal, Anirban Sinha, Anuj Kumar Pandey, Animesh Maiti

**Affiliations:** 1 Department of Endocrinology, Kasturba Medical College, Manipal, Karnataka India; 2 Department of Endocrinology & Metabolism, Medical College, Kolkata, MCH 4th floor, 88 College Street, Kolkata, West Bengal 700073 India; 3 Department of Paediatrics, King George’s Medical University, Lucknow, Uttar Pradesh India

**Keywords:** Primary hypothyroidism, Levothyroxine, Proton pump inhibitor, Pantoprazole, Thyroid function test

## Abstract

**Background:**

One of the common causes of suboptimal control of thyroid stimulating hormone (TSH) in levothyroxine-treated hypothyroidism is coadministration of proton pump inhibitors (PPIs). Morning administration of pantoprazole has been shown to suppress intragastric pH to a greater extent. We therefore aimed to determine the effect of pantoprazole at different time points of the day on thyroid function test (TFT) in levothyroxine-treated overt primary hypothyroidism.

**Methods:**

In this single centre, hospital based, prospective, two arm cross-over study (AB, BA), participants were randomized into 2 groups based on morning (6:00 am – 7:00 am simultaneously with the scheduled levothyroxine tablet) (group M) and evening (30 min before dinner) intake of 40 mg pantoprazole tablet (group N). After the initial 6 weeks (period 1), a washout period of 1 week for pantoprazole was given, and then both the groups crossed over for another 6 weeks (period 2). Patients were instructed to continue the same brand of levothyroxine tablet at empty stomach 1-hour before breakfast. Serum TSH was measured at baseline, week 6, and week 13.

**Results:**

Data from 30 patients, who completed the study with 100% compliance, were analysed. Mean TSH values of the study participants were significantly higher both at week 6 and week 13 compared to the baseline. Mean baseline serum TSH concentrations for groups M and N were 2.70 (± 1.36), and 2.20 (± 1.06) µlU/mL, respectively. Mean serum TSH concentrations at the end periods 1 and 2 for group M were 3.78 (± 4.29), and 3.76 (± 2.77) while the levels in group N were 3.30 (± 1.90), and 4.53 (± 4.590) µlU/mL, respectively. There was a significant rise in serum TSH concentration across periods 1 and 2 in both the groups (F_2, 58_ = 3.87, p = 0.03). Within group changes in TSH across periods 1 and 2 were not statistically significant. Similarly difference in TSH between the groups, either at 6 weeks or at 13 weeks, were also not statistically significant.

**Conclusions:**

Concomitant use of pantoprazole, even for 6 weeks, leads to significant elevation in serum TSH in levothyroxine-treated patients who are biochemically euthyroid, irrespective of timing of pantoprazole intake. Early morning and night-time administration of pantoprazole have similar effect on TFT in these patients.

**Supplementary Information:**

The online version contains supplementary material available at 10.1186/s13044-023-00156-6.

## Introduction

Thyroid diseases are often encountered in clinical practice, and it has been estimated that about 42 million people in India suffer from such disorders. Overall prevalence of primary hypothyroidism, in a population-based study conducted in 2013 involving more than five thousand adult subjects across 8 major cities of India, was 10.95% with Kolkata, West Bengal having the highest (21.67%) [1]. Currently, the mainstay of management of hypothyroidism, irrespective of aetiology, is oral administration of levothyroxine sodium (LT4). More than half of the LT4-treated patients in India have serum thyroid stimulating hormone (TSH) above the target range [2]. Non-compliance, inadequate dosing, inappropriate timing of LT4 administration and use of co-medications are the common reasons of elevated TSH in these patients [3]. Ingested LT4 tablets undergo disintegration and dissolution in the acidic environment of the stomach and are subsequently absorbed from jejunum and ileum. Approximately 60–82% of the total dose is absorbed within 3 hours of administration [4]. A prerequisite for adequate absorption of LT4 tablet is its active ingredient being in aqueous solution within stomach, which is further determined by intra-gastric pH and nature of excipients, amongst others. In-vitro studies, performed at 25 °C, have shown that this aqueous solubility decreases as pH increases from 1 reaching a nadir between 3 and 7, and then increases as the pH increases further [5]. The median fasting intra-gastric pH in adults varies between 1.5 and 1.7 during early morning. Any factor that raises intra-gastric pH, may also alter the ionization status and conformational characteristics of the LT4 molecule, thereby interfering with efficiency of intestinal absorption of the hormone [6].

Proton pump inhibitors (PPIs) are used to treat a variety of gastro-intestinal conditions like erosive esophagitis due to gastroesophageal reflux disease (GERD), hypersecretory conditions including Zollinger Ellison syndrome, Helicobacter pylori infection, non-steroidal anti-inflammatory drugs (NSAIDs)-induced ulcers, prevention of re-bleeding in peptic ulcers, and prophylaxis for stress ulcers in critically ill patients. They are even more frequently used as an over the counter (OTC) drug for dyspepsia across the world, including India [7]. Since gastrointestinal manifestations like dyspepsia or nausea due to delayed gastric emptying are not uncommon in patients with hypothyroidism, PPIs are often prescribed to them [8, 9]. PPIs increase intragastric pH and can potentially impair absorption of orally administered LT4 tablet. The precise prevalence of PPI use in patients with hypothyroidism is largely unknown; however, retrospective studies like the Thyroid Epidemiology, Audit and Research Study (TEARS) or data extracted from the Italian general practice Health Search CSD Longitudinal Patient Database (HSD) found that PPI co-prescription varies from 8 to 69.8% in LT4-treated subjects [10, 11].

Effect of PPI on gastric acid suppression depends to some extent on time of the drug intake. In a randomized, double-blind, two-period crossover comparative study, significantly greater increase in 24-hour median intra-gastric pH was noted following pre-breakfast (8:00 AM) administration of 40 mg pantoprazole compared to pre-dinner (7:00 PM) administration (3.3 vs. 2.7) [12]. There are handful of prospective studies that looked into the alteration in thyroid function test (TFT) in patients being treated with both LT4 tablet and one of the PPIs. However, none of these studies or the other retrospective studies evaluated the effect of timing of administration of PPI on TFT in LT4-treated patients [6, 13, 14]. We hypothesized that absorption of orally administered LT4 tablet is variably altered if PPI is taken in the morning versus at night. The objectives of this study were (A) to assess the effect of co-prescription of pantoprazole on TFTs in patients with overt primary hypothyroidism being treated with LT4, and (B) to determine the effect of pantoprazole, administered at different time points of the day, on TFTs in the same cohort.

## Materials and methods

**Fig. 1:**
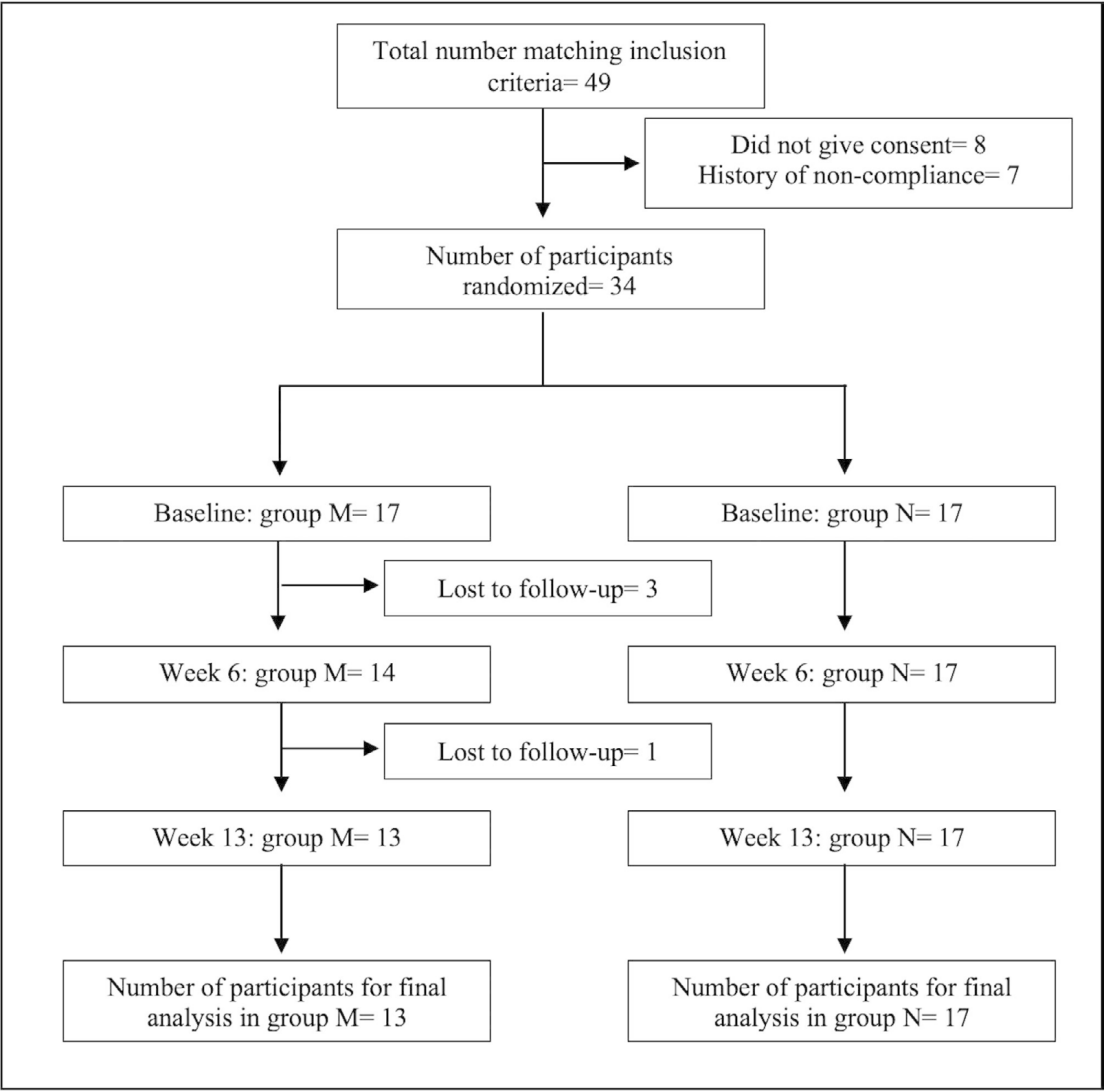
Patient flow diagram

**Fig. 2:**
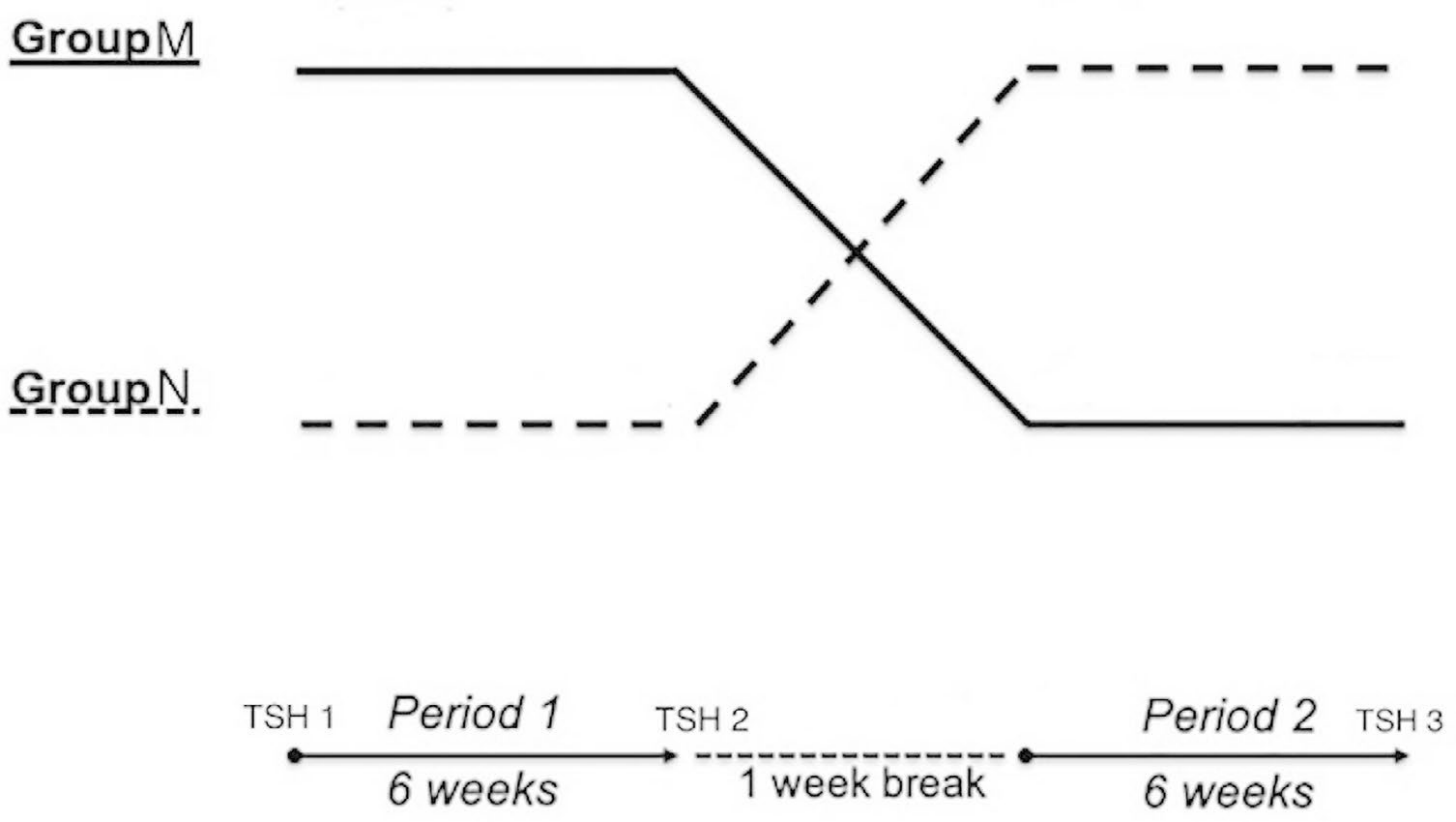
Study design

This was a single centre, hospital based, prospective, two arm cross-over study (AB, BA). The study was performed in the Department of Endocrinology & Metabolism, Medical College, Kolkata, India between February 2020 to July 2022 after approval of the Institutional Ethics Committee (Ref. No. MC/KOL/IEC/NON-SPON/701/03/2020). During the initial screening visits, all patients attending the out-patient department, being treated with LT4 following a diagnosis of overt primary hypothyroidism [low free thyroxin (FT4) and TSH of at least 20 µIU/mL at diagnosis] were identified. Patients with history of depression or other psychiatric disorders, pituitary disorders, cirrhosis, chronic kidney disease, nephrotic syndrome, malabsorption, underlying malignancy, recent hospitalization or corticosteroid use; those receiving medications known to affect thyroid hormone metabolism or absorption during preceding 3 months (PPI, H2 receptor blocker (H2RB), orlistat, ciprofloxacin, ferrous sulphate, calcium supplementation, rifampicin, raloxifene, sevelamer, sucralfate, polystyrene sulfonate, bile acid sequestrants, tyrosine kinase inhibitors, glucocorticoids) were excluded. Among the eligible candidates, those with a documented past history of noncompliance or those who were unwilling or could not give consent were also excluded. From the remaining population, adult subjects (age between 18 and 70 years) who were biochemically euthyroid (FT4 between 0.8 and 1.76 ng/dL and TSH between 0.4–4.78µIU/mL) and had stable TSH level (variation not more than 1.6 µIU/mL or 40%, whichever is lower) for at least 6 months prior to recruitment on a fixed dose of LT4 (no change in dose or brand for at least 6 months) were then randomized into two groups (M and N) using random allocation table generated by MS Excel by AM (one of the authors) who was blind to the intervention type (Fig. 1). M group received morning dose (between 6:00–7:00 AM) of oral pantoprazole at empty stomach for 6 weeks (along with scheduled LT4 1-hour before breakfast), and N group received a night dose of oral pantoprazole for 6 weeks (30 minutes before dinner). TSH was repeated after the initial 6 weeks and pantoprazole was withheld for one week. Patients in the M group were then crossed over and took pantoprazole 30 minutes before dinner, and those in the N group were crossed over and took pantoprazole 40 mg in the morning for the next 6 weeks. TSH was rechecked again at that point of time (week 13) (Fig. 2).

The same brand of LT4 tablet was continued throughout the study period, dose being the same as the one being used for the preceding 6 months. In both the groups (M and N), LT4 was consumed immediately after waking up at empty stomach as the first thing in the morning and nothing was allowed for 1 hour except water. We used 40 mg of pantoprazole of the same brand (Pantodac® 40, Zydus Corza, Zydus Healthcare Ltd. Unit-II, East-Sikkim, India). Both LT4 and pantoprazole were consumed under direct supervision of a family member. A compliance/adherence chart was given to each of the participants, which was needed to be filled up with administrations of both the drugs. For facilitating adherence patients were communicated daily through WhatsApp® along with weekly telephonic reminders. Good adherence was intake of 100% of the scheduled drugs (LT4 and PPI) on the designated time. Primary outcome measure was serum TSH concentration, measured after drawing 5 ml of peripheral venous blood in red-capped vial thrice during the study, first at baseline (TSH1), then at week 6 (± 2 days) after completing period 1 (TSH2), and the third at week 13 (± 2 days) corresponding to the end of period 2 (TSH3).

### Laboratory methods

TFT was obtained from morning samples after an overnight fast before the scheduled morning dose of LT4. FT4 and TSH assays were performed by automated direct chemiluminescence method on ADVIA Centaur XP platform (TSH3-Ultra assay, Siemens Healthcare Diagnostics, Erlangen, Germany) using kits of a single batch. The assay range for the TSH3-Ultra assay was from limit of quantification (functional sensitivity: 0.008 µIU/mL) to 150 µlU/mL with intra-assay variation of 1.4–2.4% and inter-assay imprecision of 0.9–2.9%. The intra-assay and inter-assay coefficient of variation (CV)s for FT4 were 2.2–3.3% and 2.5-4.0%, respectively. The reference intervals for TSH and FT4 were 0.4–4.78µIU/mL and 0.8–1.76 ng/dL, respectively.

### Statistical methods

The calculated sample size was 28 (for a level of significance 5% and power of 80%) (vide supplementary file). Presuming a dropout rate of at least 10%, we recruited 34 eligible subjects, 17 in each group. 4 participants in group M did not complete the study, hence data from 30 patients, 13 in group M and 17 in group N were finally analysed.

Data were collected using a pretested questionnaire and entered in MS Excel. For statistical analysis, IBM SPSS Statistics for Windows, Version 20.0. was used (IBM Corp. Released 2011. IBM SPSS Statistics for Windows, Version 20.0. Armonk, NY: IBM Corp.). Data were summarized as mean and standard deviation (SD) for numerical variables with normal distribution. A paired t-test was used to compare overall mean TSH at baseline and at week 6 (TSH2 vs. TSH1), and at baseline and at week 13 (TSH3 vs. TSH1). A p value of < 0.05 was taken as statistically significant using a two-tailed distribution. Repeated measure ANOVA test was used to assess the mean TSH concentration (primary outcome) in participants before and after using pantoprazole. F-value with degrees of freedom was calculated. A p-value of < 0.05 was taken as statistically significant using a two-tailed distribution. ANOVA were then analysed using post hoc tests to establish which changes in mean TSH concentrations were significant. Bonferroni corrected p-value for post-hoc test was calculated. Paired t-test was used to compare the changes in mean TSH concentration between initial 6 weeks and subsequent 6 weeks separately for group M and group N. An unpaired t-test was used to compare the changes in TSH concentration between group M and group N. The changes in TSH from baseline at end of week 6 (TSH2-TSH1), and change of TSH from week 6 to week 13 (TSH3-TSH2) was used for both the paired and unpaired t-tests.

Mann Whitney U-test was utilized to compare the median changes in serum TSH concentrations in those who took pantoprazole in the morning (TSH2-TSH1 in group M, and TSH3-TSH2 group N) with those who took pantoprazole at night (TSH3-TSH2 in group M, and TSH2-TSH1group N). A p value of < 0.05 was considered significant.

Post-hoc analysis to look for ‘clinically meaningful’ elevation of TSH at the ends of each treatment phase were dichotomized to any significant elevation and no change/decrement, and between-group treatment differences were determined using McNemar’s test with Yates correction. A p value of < 0.05 was taken as statistically significant. The results were then rechecked and represented appropriately for better understanding.

## Results

From February 2020 to July 2022, a total of 34 cases were recruited for the study. Four participants in the M group did not come for subsequent follow-up due to prevailing SARS-Cov2 pandemic, hence per-protocol analysis was done using data from 30 patients with 100% adherence and complete follow-up. The mean age for the study population was 38.57 (± 12.56) years and 76.7% (n = 23) were women. The mean duration of primary hypothyroidism was 6.01 (± 4.86) years. The baseline characteristics of the study population have been summarized in Table 1 (and supplementary file-Table 1).

**Table 1:** Descriptive statistics of demographics and thyroid function tests among groups at baseline

Characteristics of variables	Group M N = 13	Group N N = 17	p-value
	mean ± SD		
Age (years)	37.23 ± 11.28	39.59 ± 13.71	0.62
Female, n (%)	12 (92.3)	11 (64.7)	0.18
BMI (Kg/ m^2^)	26.71 ± 5.16	24.92 ± 4.76	0.33
Duration of disease (years)	5.08 ± 3.01	6.88 ± 5.87	0.32
FT4 (ng/ dL)	1.22 ± 0.28	1.34 ± 0.30	0.32
TSH (µIU/mL)	2.70 ± 1.36	2.20 ± 1.06	0.27
LT4 dose (µg/ day)	88.58 ± 24.18	85.35 ± 28.73	0.75

**Fig. 3:**
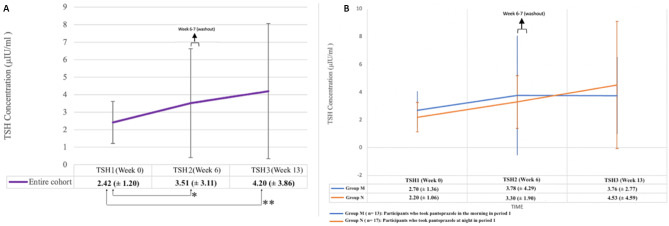
Mean TSH concentration of the entire cohort (n = 30) at different time points during the study **(A)** and mean TSH concentration in groups M (n = 13) and N (n = 17) at different time points during the study **(B)** (week 6–7 is the washout period where participants were off pantoprazole; week 7: cross-over)

Baseline TSH was normally distributed (skew = -0.06) and platykurtic (-1.16). The mean TSH concentrations of all the study participants (n = 30) at baseline (TSH1), week 6 (TSH2), and at week 13 (TSH3) were 2.42 (± 1.2) µIU/mL, 3.51 (± 3.11) µIU/mL, and 4.2 (± 3.86) µIU/mL, respectively. Mean changes in TSH concentrations of all the study participants at week 6 from baseline (TSH2 – TSH1), and at week 13 from baseline (TSH3-TSH1) were 1.09 (± 3.33) µlU/mL (p = 0.04) and 1.78 (± 4.05) µIU/ mL (p = 0.01), respectively (Fig. 3A). There was a rise in TSH of 35.53% in the entire cohort at the end of week 13.

Mean TSH1 for group M and group N was 2.70 (± 1.36) µlU/mL and 2.20 (± 1.06) µlU/mL, respectively. Mean TSH2 in group M and group N were 3.78 (± 4.29) µlU/mL and 3.30 (± 1.90) µlU/mL, and mean TSH3 were 3.76 (± 2.77) µlU/mL 4.53 (± 4.59) µlU/mL, respectively (Table 2; Fig. 3B). There was a significant rise in TSH concentrations across the study period (F_2, 58_ = 3.87, p = 0.03). Post hoc tests revealed significant difference between TSH1 and TSH3 concentrations (p = 0.04) (Table 2). However, changes between TSH2 and TSH1, and between TSH3 and TSH2 were not significant (Table 2).

**Table 2:** TSH concentrations in three different time periods

Groups	TSH1	TSH2	TSH3
	Mean (± SD)	Mean (± SD)	Mean (± SD)
Total study population (n = 30)	2.42 (± 1.20)	3.51 (± 3.11)	4.20 (± 3.86)
Group M (n = 13)	2.70 (± 1.36)	3.78 (± 4.29)	3.76 (± 2.77)
Group N (n = 17)	2.20 (± 1.06)	3.30 (± 1.90)	4.53 (± 4.59)

Repeated measure ANOVA shows a significant effect for the within-subject factor “time” (F2, 58 = 3.87, p = 0.03). Post hoc-test shows the following p values: TSH1 vs. TSH2 (p = 0.13), TSH2 vs. TSH3 (p = 1.0) and TSH1 vs. TSH3 (p = 0.04).

The mean change in serum TSH (TSH2-TSH1) after consumption of morning dose of pantoprazole for initial 6 weeks in group M was 1.07 (± 3.89) µlU/mL. After the end of study period, i.e. after 6 weeks of bed time pantoprazole use, the mean change in TSH (TSH3-TSH2) in this group was − 0.01(± 4.08) µlU/mL. There was no statistical significance between the changes in TSH during these two periods (p = 0.61). In the N group, the mean changes in TSH after 6 weeks (following bed time pantoprazole use) and after 13 weeks (following morning dose of pantoprazole) were 1.10 (± 1.70) µlU/mL and 1.22 (± 3.93) µlU/mL, respectively. Once again, the changes during these two periods were not statistically significant (p = 0.91) (Table 3). The changes in TSH between the two groups during the initial 6 weeks (p = 0.98) and the subsequent 6 weeks (p = 0.40) were also insignificant (Table 3). The mean changes in TSH from baseline to the end of the study, i.e., TSH3-TSH1 were 1.06 (± 3.09) µlU/mL, 2.33 (± 3.21) µlU/mL in the M group and N group, respectively.

**Table 3:** Changes in TSH concentration in both the groups with relation to timing of pantoprazole intake

Groups	TSH2-TSH1	TSH3-TSH2	TSH3-TSH1	p
	Mean (± SD)	Mean (± SD)	Mean (± SD)	
Study population (n = 30)	1.09 ± 3.33	0.69 ± 4.96	1.78 ± 4.05	
Group M (n = 13)	1.07 ± 3.89	-0.01 ± 4.08	1.06 ± 3.09	0.61*
Group N (n = 17)	1.10 ± 1.70	1.22 ± 3.93	2.33 ± 3.21	0.91*
p	0.98**	0.40**	0.36**	

* Paired t-test between TSH2-TSH1 and TSH3-TSH2 in Group M and Group N

** Unpaired t-test between Group M and Group N for TSH2-TSH1, TSH3-TSH2 and TSH3-TSH1

Median change in serum TSH concentrations with morning administration of pantoprazole (TSH2-TSH1 in group M, and TSH3-TSH2 in group N) was 0.21 (− 0.98, 1.14), and with night-time administration (TSH3-TSH2 in group M, and TSH2-TSH1 in group N) was 0.69 (− 0.57, 1.99). The difference was statistically insignificant (p = 0.40).

We also looked at ‘clinically meaningful’ changes, as defined by absolute value of TSH more than 4.78 µIU/mL, upper limit of our reference range, or an increase of TSH by more than 1.6 µIU/mL and/or more than 40% from baseline. 26.7% patients (n = 8) had TSH of more than 4.78 µIU/mL following initial 6 weeks of pantoprazole use. Among these 8 patients, 3 (23%) patients were in group M and 5 (29.4%) were in group N. After the end of study period, 33.3% (n = 10) had TSH of more than 4.78 µIU/mL of which 6 were in group M (46.1%), and 4 (23.5%) in group N. When we looked at the changes in TSH from baseline, a total of 12 (40%) patients (Group M: 4, Group N:8) had noticed TSH elevation of more than 1.6 µIU/mL and/or more than 40% after 6 weeks and 15 (50%) patients (Group M: 6, Group N: 9) had similar changes at the end of the study. However, such ‘clinically meaningful’ changes in TSH that we found in our study were not statistically significant in either groups both at week 6 and week 13 (McNemar’s test with Yates correction = 0.19, p = 0.67).

## Discussion

The mean age of participants in our study was 38.57 (± 12.56) years with a mean duration of hypothyroidism of 6.01 (± 4.86) years suggesting that the study participants were already on LT4 for a substantial period of time before recruitment. There was a female gender preponderance in our study group that conforms to the results from the Indian prevalence data showing higher prevalence of hypothyroidism in females as compared to males [1]. The gender distribution is also consistent with other studies which have looked at the prevalence of hypothyroidism [15]. Though a combination of subnormal FT4 and elevated TSH, usually above 10 µIU/mL, characterizes overt primary hypothyroidism, we took a TSH cut-off of 20 µIU/mL as TSH may increase up to 20 µIU/mL during the recovery phase of thyroiditis or non-thyroidal illnesses.

Injudicious PPI use is exceedingly common in clinical practice either prescribed by general physicians or as OTC agent, particularly for functional dyspepsia [16]. In India, pantoprazole is the most commonly prescribed PPI [17]. Of the available PPIs, lansoprazole and omeprazole have been found to interfere with LT4 absorption, and thus increase TSH in individuals who are biochemically euthyroid on stable dose of LT4. Two retrospective studies have documented a median increase of TSH by 0.69 (± 1.9) µIU/mL and 0.18 µIU/mL after 2–6 months of PPI intake in such patients (p = 0.035 and 0.001, respectively) [11, 18]. Retrospective review of the Italian general practice HSD also showed increased TSH levels with concomitant PPI intake (adjusted incidence ratio rate: 1.02; 95% CI: 1.01–1.03) [10]. Participants in our study were given daily PPI for initial 6 weeks and then for another 6 weeks following a washout period of 1 week. With morning dose of pantoprazole TSH rose from 2.70 µIU/mL to 3.78 µIU/mL, and with night dose TSH increased from 2.20 µIU/mL to 3.30 µIU/mL during the initial 6 weeks. We documented that daily intake of PPI even for 6 weeks also lead to significant impairment in LT4 absorption irrespective of time of administration, as evidenced by a TSH elevation of 1.07 µIU/mL in group M, 1.10 µIU/ mL in group N and 1.09 µIU/ml in the entire study population. With longer duration of PPI therapy (12 weeks), the changes were even more pronounced. The mean increment in TSH from baseline was 1.06 µIU/mL in M group, 2.33 µIU/mL in N group and 1.78 µIU/mL overall at the end of the study (Table 2; Fig. 3A and B).

Co-prescription of LT4 and usual dose of PPI also leads to clinically significant elevation in TSH due to inadequate LT4 absorption, which resulted in 35–37% higher dose of LT4 to achieve target TSH in different cohorts of patients with euthyroid nodular thyroid disease or hypothyroidism [6, 18]. However, we did not look into the percentage increase in LT4 dosage to achieve biochemical euthyroidism in our patients.

The effect of PPIs on LT4 absorption, however, has been discordant. While some studies documented reduced absorption of LT4 tablet with lansoprazole (30 mg daily), omeprazole (40 mg per day) or intravenous esomeprazole (80 mg) [6, 18, 19], others did not find similar effects with omeprazole, esomeprazole or pantoprazole. Potential dose dependent reduction in LT4 absorption with two different doses of omeprazole (20 mg and 40 mg) were studied in primary hypothyroid subjects who were biochemically euthyroid on LT4 tablets for at least 1 year (n = 19). There was no statistically significant difference in TSH levels before and 3 months after 20 mg (median levels: 2.24 vs. 2.42 µIU/ml, p = 0.62) or 40 mg (median level: 2.28 and 2.30 µIU/mL, p = 0.82) of omeprazole therapy [20]. Similar observation was noted in serum thyroid hormone concentrations after 600 µg of LT4 administration following 1-week therapy with esomeprazole (and famotidine) [21]. However, this study was biased by a short sampling time (8 hour), which is much lower than the half-life of LT4, making a reliable assessment of the pharmacokinetic profile somewhat difficult. The differences in dose, route of administration or in PPI molecule might explain, at least partly, this discrepancy in TSH changes. Though gastric acid suppression is attributed to a class effect of PPIs, differences in potency and pharmacokinetic profile among different molecules may lead to variable effect on LT4 absorption. We used a single dose of 40 mg of pantoprazole. A crossover study involving 20 healthy subjects, however, found that pantoprazole had no effect on LT4 absorption. TSH was measured following a single dose of LT4 (4 µg/kg) at baseline and after 1 week of 40 mg pantoprazole therapy [13]. An important drawback of both these studies (esomeprazole & pantoprazole) was the short course (1 week) of PPI therapy. Steady state is usually achieved after 4–6 weeks of LT4 therapy; hence, any alteration in circulatory TSH concentration may not become apparent after 1 week of PPI therapy. Though a plateau in gastric acidity has been described following 4 days of PPI use in some studies, and suppression of gastric acid secretion was documented through increased gastrin level in the pantoprazole study, the investigators used a very high dose of LT4 (4 µg/kg), which is much higher than the usual dose required in athyreotic individual (1.6–1.8 µg/kg/day) [13]. Such an unusual high dose may overcome PPI interference on LT4 absorption. Long-term PPI treatment is likely to alter gastrointestinal transit time, mucosal architecture, and local milieu along with alteration in enterohepatic metabolism of orally administered LT4. The other potential confounders of this study were short sampling time (10 hour), which is insufficient to alter TSH, and to accurately determine T4 kinetics profile, and dose of LT4; a dose of 600 µg (which is much higher than 4 µg/kg) is needed for accurate assessment of LT4 pharmacokinetics and precise discrimination in potential differences in LT4 absorption. We did not find any difference between time of administration of pantoprazole as far as increase in serum TSH were concerned (Table 3). Serum TSH concentrations were equally elevated both with morning and bed time dose of pantoprazole.

Oral pantoprazole has a bioavailability of 77% and its absorption is not affected by food or antacids. It is absorbed from the small bowel, resulting in a maximum serum concentration 2 to 3 hours post-ingestion. Unlike other PPIs, the serum concentration of pantoprazole is not dose-dependent; serum concentration after the first dose is similar to that following multiple doses. The metabolism of pantoprazole is independent of route of administration, with a half-life of approximately 1 hour [22]. Gastric acid suppression is a class effect of the PPIs. Though the half-life of the available PPIs (other than tenatoprazole) is 1–2 hours, the duration of acid inhibition is almost 48 hours because of irreversible binding to the H^+^K^+^-ATPase [23]. Once the steady sate is achieved, the duration of action of pantoprazole lasts for about a week. Duration of acid suppression with ranitidine, a H2RB is 4–6 hours. LT4-treated patients, who require gastric acid suppressive therapy, thus, should better be offered H2RB after 3–4 hours of LT4 administration to avoid elevation in TSH. PPI use even for 6 weeks, irrespective of timing of administration, is likely to result in increment in ongoing LT4 dosage to maintain the TSH level.

Intra-individual fluctuation in serum TSH is physiological and differences in TSH values, measured in two different time points does not necessarily indicate abnormal test result. Individual reference ranges of TSH is much narrow than the population-specific reference intervals, and it has been suggested that changes in serum TSH of as low as 0.75µIU/ml (range: 0.2–1.6 µIU/ml) may take a particular individual out of their ‘personal reference range’ [24]. However, a ‘true difference’ between two TFTs is said to be present if there is 40% variation in TSH and 15% in FT4 [25]. It needs to be remembered that these cut-offs were derived from longitudinal follow-up of patients with stable untreated subclinical hypothyroidism for 1 year. The intra-individual variations in TFT in patients with LT4-treated overt hypothyroidism within 13 weeks are expected to be much lower. Following lansoprazole initiation, 19% patients noticed TSH of more than 5 µIU/mL, which necessitated LT4 dose increment [18]. In our cohort, TSH went up the upper limit of the reference range (and also more than 5µIU/ml) in 26.7% after 6 weeks, and in 33.3% after 12 weeks of pantoprazole therapy. In addition, a large proportion of our cohort (40% at 6 weeks and 50% at end of study) had increment in TSH, beyond the upper limit of expected intra-individual variation, described earlier. Though the findings were not statistically significant, our study was not powered to look for such changes in serum TSH concentrations. Duration of PPI therapy could possibly result in ‘clinically meaningful’ elevation in serum TSH as well.

Interestingly, absorption of LT4 formulations, other than tablet, seems unaffected by PPIs. LT4 absorption kinetics were assessed following intravenous esomeprazole (80 mg), and coadministration of 600 µg of LT4 either in tablet form or in gel capsule form [19]. Under esomeprazole infusion, Cmax of LT4 decreased by 13.0% with the tablet, but only 2.6% with the gel capsule. Gelatine coating of the gel capsules possibly helped in absorption and obliviated the need for low intra-gastric pH [19].

Compared to tablets, absorption of liquid formulation of LT4 seems to be unaffected by simultaneous PPI use. In an earlier study, LT4 dose requirement decreased when patients were switched to liquid formulation from tablets while on any of the 4 different PPI molecules (omeprazole, pantoprazole, lansoprazole, esomeprazole) [14]. As discussed earlier, aqueous form of LT4 is required for its effective absorption. Impaired gastric acid secretion following PPI use interfere with the aqueous formation of administered LT4 tablets resulting in reduced absorption. Gel-capsule and liquid formulations of LT4 do not require low intra-gastric pH for absorption, hence unaffected by coadministration of PPI. However, these formulations are not widely available.

This study was conducted in a prospective, 2-arms, randomized, cross-over pattern, which is inherently a strong study design. To the best of our knowledge, this is the first prospective study in the literature, which assessed the effect of PPI administration in two different point of times on change in TSH concentration in a cross-over pattern. We opted for a very strict inclusion criteria as far as variation in TSH was concerned (variation not more than 1.6 µIU/mL or 40% whichever is lower for at least 6 months). The baseline characteristics were similar between both the groups. Furthermore, medications which are known to affect LT4 absorption or metabolism were excluded; thus, removing any potential confounder. We ensured 100% compliance both for PPI and LT4. In addition, we tried to look at ‘clinically meaningful’ changes in serum TSH concentrations with PPI use.

Our study had certain limitations. Due to the Covid-19 pandemic, we could recruit only 30 patients. This may have been enough to render the study adequately powered; however, more number of participants should have been enrolled to provide further strength. Four patients were lost to follow-up in group M, which led to a female gender predominance in group M. This was addressed by assessing the changes in TSH between the two groups after removing all male participants in both the groups. The results from this analysis were concordant with our overall findings. The study participants were under strict vigilance of the investigators and their relatives to ensure compliance with study medications (LT4 and pantoprazole). This may have inadvertently led to the Hawthorne effect leading to enhanced adherence. This also explains decreased in TSH concentration from baseline in some of the participants. Such strict adherence is often not seen in clinical practice. A placebo arm to the cross-over design could have further strengthened the study. However, we could not arrange for ‘matching placebo’ tablets. One potential limitation might be a short wash-out period of 1 week for pantoprazole. The duration of acid inhibition following PPI use is 48 hours; one week, thus, seems sufficient for wash-out [23]. Another limitations of the study is single centre recruitment of patients. We did not assess anti-thyroid antibodies in the study participants. Patients with chronic lymphocytic thyroiditis may require progressive increment of LT4 doses during follow-up, as remaining thyroid function gradually declines. However, the duration of hypothyroid was 6 years before recruitment and participants had stable TSH during the preceding 6 months. It is unlikely that the residual gland function declined during that 13 weeks of the study resulting in increased TSH with stable dose of LT4.

## Conclusion

Concomitant use of pantoprazole and LT4 tablets leads to significant elevation of serum TSH concentration in LT4-treated hypothyroid patients who are biochemically euthyroid. Such increment in TSH is encountered even after 6 weeks of co-therapy and develops irrespective of timing of pantoprazole use, either with early morning or with bed time administration. The degree of increment in TSH does not differ between early morning or night-time use of pantoprazole. We noticed a trend in ‘clinically meaningful’ increase in TSH with pantoprazole use. Though, statistically insignificant in our study, increase in TSH might be ‘clinically meaningful’ in a group of patients, and further studies with larger cohort are needed to look at such changes in serum TSH concentrations with PPI co-prescription.

## Electronic supplementary material

Below is the link to the electronic supplementary material.

**Supplementary Material 1**: Calculation of sample size and characteristics of individual participants and their TSH values

## Data Availability

Authors are ready to produce all relevant data, if required at any point of time. The data that support the findings of the study are available on request from the corresponding author. The data are not publicly available due to privacy or ethical restrictions.

## Abbreviations

BMI: Body mass index
FDA: Food and Drug Administration
FT4: Free tetraiodothyronine
GERD: Gastroesophageal reflux disease
GI: Gastrointestinal
H2RB: H2 receptor blocker
LT4: Levothyroxine sodium
NSAID: Non-steroidal anti-inflammatory drug
OTC: Over the counter
PPI: Proton pump inhibitor
SARS-Cov2: Severe acute respiratory syndrome coronavirus 2
SD: Standard deviation
T3: 3,5,3′-l-triiodothyronine
T4: 3,5,3′5′-l-tetraiodothyronine
TFT: Thyroid function test
TPO: Thyroid peroxidase
TSH: Thyroid stimulating hormone
μIU: Micro international unit
μg: Microgram
