# Supplementary material for: Effect of morning versus night-time administration of proton pump inhibitor (pantoprazole) on thyroid function test in levothyroxine-treated primary hypothyroidism: a prospective cross-over study
Source: Thyroid Res. 2023 Jun 1;16:15. doi: 10.1186/s13044-023-00156-6 (PMC10231962; doi:10.1186/s13044-023-00156-6)
Supplement: Supplementary file 1 — Supplementary Material 1: Calculation of sample size and characteristics of individual participants and their TSH values [file 13044_2023_156_MOESM1_ESM.docx]

**Data 1: Calculation of sample size**

Level of significance = 5%, Power = 80%, Type of test = two-sided

Formula of calculating sample size is

n = [(Zα/2 + Zβ)2 × {2(ó)2}]/ (μ1 - μ2)2

n = sample size required in each group,

μ1 = mean change in TSH from baseline to week 4 on concomitant PPI intake = 0.69,

μ2 = mean change in TSH from baseline to week 4 without PPI = 0.11,

μ1-μ2 = clinically significant difference = 0.58 (19)

ó = standard deviation = 1.195

Zα/2: This depends on level of significance, for 5% this is 1.96

Zβ: This depends on power, for 80% this is 0.84

Calculated sample size is 28

**Supplementary Table 1: Characteristics of individual participants in the study**

| Serial number | Age  (years) | Sex | Duration of hypothyroidism  (years) | LT4 dosage  (μg) | FT4  (ng/ dL) | TSH1  (μIU/ mL) | TSH2  (μIU/ mL) | TSH3  (μIU/ mL) |
| --- | --- | --- | --- | --- | --- | --- | --- | --- |
| M1 | 40 | Female | 3 | 75 | 1.15 | 2.19 | 1.45 | 5.06 |
| M2 | 34 | Male | 4 | 125 | 1.34 | 0.56 | 0.68 | 0.59 |
| M3* | 31 | Female | 3 | 50 | 0.86 | 4.9 |  |  |
| M4 | 34 | Female | 2 | 88 | 1.27 | 3.91 | 16.61 | 5.58 |
| M5* | 27 | Male | 4 | 125 | 1 | 3.01 |  |  |
| M6 | 30 | Female | 8 | 75 | 1.31 | 3.98 | 1.99 | 6.15 |
| M7 | 26 | Female | 6 | 88 | 0.92 | 3.9 | 4.3 | 3.33 |
| M8 | 55 | Female | 9 | 50 | 1.25 | 1.96 | 6.81 | 8.28 |
| M9 | 23 | Female | 11 | 100 | 1.57 | 2.78 | 0.97 | 0.19 |
| M10 | 38 | Female | 3 | 50 | 1.14 | 4.65 | 5.29 | 4.62 |
| M11 | 52 | Female | 5 | 112.5 | 1.76 | 1.48 | 0.78 | 6.75 |
| M12 | 25 | Female | 7 | 100 | 0.95 | 4 | 4.19 | 5.42 |
| M13 | 27 | Female | 2 | 75 | 1.55 | 1.57 | 2.36 | 0.82 |
| M14 | 49 | Female | 5 | 125 | 0.85 | 3.34 | 2.09 | 0.04 |
| M15 | 51 | Female | 1 | 88 | 0.91 | 0.81 | 1.58 | 2.11 |
| M16* | 58 | Male | 6 | 37.5 | 1.52 | 2.43 |  |  |
| M17* | 18 | Female | 9 | 75 | 1.58 | 2.83 | 3.93 |  |
| N1 | 19 | Female | 7 | 88 | 1.67 | 3.54 | 3.73 | 2.77 |
| N2 | 30 | Male | 9 | 75 | 1.22 | 2.27 | 2.91 | 1.73 |
| N3 | 45 | Female | 20 | 75 | 0.87 | 3.19 | 5.57 | 4.03 |
| N4 | 49 | Male | 18 | 75 | 1.2 | 1.97 | 1.47 | 6.24 |
| N5 | 38 | Female | 1 | 100 | 1.53 | 3.21 | 5.07 | 10.95 |
| N6 | 30 | Female | 4 | 150 | 1.39 | 2.91 | 5.49 | 15.15 |
| N7 | 64 | Female | 13 | 50 | 0.9 | 2.26 | 1.29 | 1.51 |
| N8 | 26 | Female | 1 | 75 | 1.01 | 2.43 | 5.39 | 14.25 |
| N9 | 31 | Female | 2 | 50 | 1.1 | 1.4 | 2.54 | 2.83 |
| N10 | 45 | Female | 3 | 50 | 1.16 | 3.34 | 4.08 | 3.04 |
| N11 | 50 | Female | 11 | 100 | 1.7 | 0.81 | 1.62 | 3.81 |
| N12 | 21 | Female | 1 | 50 | 1.03 | 3.14 | 4.14 | 4.69 |
| N13 | 36 | Male | 4 | 125 | 1.45 | 1.26 | 0.73 | 0.41 |
| N14 | 40 | Male | 6 | 112.5 | 1.72 | 3.4 | 2.94 | 2.13 |
| N15 | 70 | Male | 2 | 112.5 | 1.55 | 0.48 | 0.5 | 0.12 |
| N16 | 41 | Male | 10 | 75 | 1.43 | 1.12 | 2.01 | 2.27 |
| N17 | 38 | Female | 5 | 88 | 1.8 | 0.7 | 6.67 | 1.02 |
| *did not complete the study. Data was not included in the final analysis | | | | | | | | |

**Data 2: List of abbreviations**

BMI: Body mass index

FDA: Food and drug administration agency

FT4: free tetraiodothyronine

GERD: Gastroesophageal reflux disease

GI: Gastrointestinal

H2RB: H2 receptor blocker

LT4: Levothyroxine sodium

NSAID: Non-steroidal anti-inflammatory drug

OTC: Over the counter

PPI: Proton pump inhibitor

SARS-Cov2: Severe acute respiratory syndrome coronavirus 2

SD: Standard deviation

T3: 3,5,3’-l-triiodothyronine

T4: 3,5,3’5’-l-tetraiodothyronine

TFT: Thyroid function test

TPO: Thyroid peroxidase

TSH: Thyroid stimulating hormone

μIU: micro international unit

µg: microgram
